# Paeonol, an Ingredient of Kamishoyosan, Reduces Intracellular Lipid Accumulation by Inhibiting Glucocorticoid Receptor Activity in 3T3-L1 Cells

**DOI:** 10.3390/nu12020309

**Published:** 2020-01-24

**Authors:** Masayuki Izumi, Takashi Yoshida, Takashi Nakamura, Minoru Wakamori

**Affiliations:** Division of Molecular Pharmacology & Cell Biophysics, Department of Oral Biology, Tohoku University Graduate School of Dentistry, Sendai 980-8575, Japan; izumi-masa@umin.ac.jp (M.I.); takashi.yoshida.d5@tohoku.ac.jp (T.Y.); takashi.nakamura.d2@tohoku.ac.jp (T.N.)

**Keywords:** adipocyte, Kamishoyosan, Moutan cortex, paeonol, C/EBP-δ, glucocorticoid receptor, Kampo medicine

## Abstract

Excessive triglyceride accumulation in lipid-metabolizing tissues is associated with an increased risk of a variety of metabolic diseases. Kamishoyosan (KSS) is a Kampo composed of 10 constituent herbs, and contains moutan cortex (MC) and paeonol (PN) as the major ingredient of MC. Here, we demonstrate the molecular mechanism underlying the effect of KSS on the differentiation of mouse preadipocytes (3T3-L1 cells). KSS inhibited the accumulation of triglycerides in a dose-dependent manner in 3T3-L1 cells that were induced to differentiate into adipocytes. We also found that MC and PN were responsible for the anti-adipogenetic effect of KSS and significantly suppressed the expression of CCAAT/enhancer-binding proteins-δ (C/EBP-δ) mRNA 3 days after the induction of differentiation. Thus, PN may contribute to the anti-adipogenetic property of MC in 3T3-L1 cells. In addition, PN inhibited dexamethasone (Dex)-induced glucocorticoid receptor (GR) promoter activity. Taken together, these results suggest that PN suppresses C/EBP-δ expression by inhibiting Dex-induced GR promoter activity at the early stage of differentiation and, consequently, delays differentiation into mature adipocytes. Our results suggest that the habitual intake of Kampo-containing PN contributes to the prevention of the onset of metabolic diseases by decreasing the excessive accumulation of triglycerides in lipid-metabolizing tissues.

## 1. Introduction

Metabolic syndrome is a cluster of metabolic disorders that are associated with numerous lifestyle-related risk factors [1]. Intracellular lipid accumulation is a common feature in the pathogenesis of a variety of lifestyle-related diseases such as cardiovascular disease, fatty liver, type 2 diabetes, and dyslipidemia [2,3,4,5,6]. Prevention and control of lifestyle-related diseases is a major public health concern, especially in developed countries.

The 3T3-L1 cell line is one of the adipocyte cell models used to study lipid metabolism *in vitro* [7]. A mixture of dexamethasone (Dex), 3-isobutyl-methylxanthine (IBMX), and insulin (hereafter DMI) can efficiently differentiate 3T3-L1 cells into mature adipocytes [8]. Differentiation of adipocytes can be largely divided into the early stage and the late stage. Adiponectin is a bioactive factor secreted from differentiated adipocytes [9]. We focused on CCAAT/enhancer-binding proteins (C/EBPs), forkhead transcription factor 1 (FoxO1), and peroxisome proliferator-activated receptor-gamma (PPAR-γ) transcription factors as the markers of adipocyte differentiation as well as adiponectin. C/EBPs are a family of six gene members, among which C/EBP-δ and C/EBP-β are expressed at the early stage, and C/EBP-α or PPAR-γ are expressed at the late stage of adipocyte differentiation [10,11]. Knockdown of either C/EBP-δ or C/EBP-β (in combination or independently) suppresses the differentiation of primary embryonic fibroblasts into mature adipocytes and lipid accumulation [12]. The expression of PPAR-γ is induced in response to insulin, and it increases glucose uptake in adipocytes [13,14]. FoxO1 is a transcription factor containing characteristic a winged helix structure termed the Forkhead box. FoxO1 is involved in the commitment of the early stage of adipogenesis by insulin *via* Akt and FoxO1 phosphorylation by the insulin signaling [15].

Traditional Japanese herbal medicine (Kampo medicine) was originally based on traditional Chinese medicine but was adapted to Japanese culture [16,17]. Kamishoyosan (KSS, Chinese name: Jiaweixiaoyaosan, Korean name: Gamisoyosan) is a complex drug composed of 10 herbs. It is prescribed for climacteric disorder, dysmenorrhea, neurosis, and in cancer supportive therapy [18,19]. Hormones and cytokines released from adipocytes are involved in the aggravation of diseases for which KSS is prescribed [20,21,22,23,24]. KSS reduces lipid accumulation in human hepatoma HepG2 cells in the presence of oleic acid [25]. In Kampo medicine, Orengedokuto, inhibits differentiation of 3T3-L1 cells and Yokukansan reduces fat synthesis by reducing the expression of the transcription factor SREBP-1c and glycerol-3-phosphate dehydrogenase and increasing the expression of antioxidant enzymes *via* the transcription factor FoxO1 in differentiating 3T3-L1 cells [26,27]. One of the compounds included in KSS, geniposide, reduces lipid accumulation in 3T3-L1 cells during differentiation [28]. Another compound, paeonol, reduces lipid accumulation in HepG2 cells [29].

The molecular mechanism underlying the pharmacological action of KSS in 3T3-L1 cells during their differentiation into mature adipocytes is unclear. Here, we demonstrate that paeonol is an inhibitory compound of KSS during adipogenesis.

## 2. Materials and Methods

### 2.1. Cell Culture

**Figure 1:**
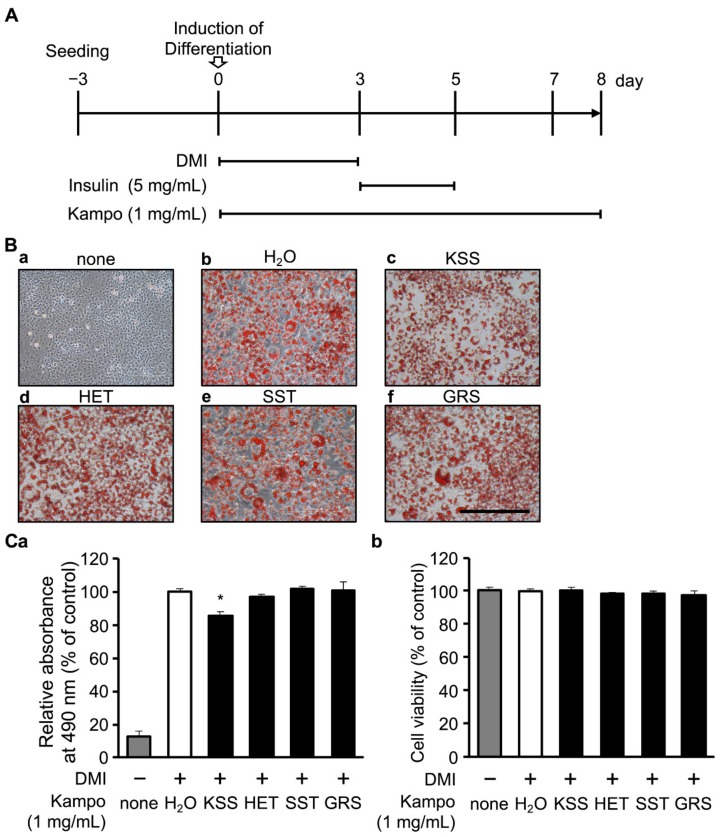
Kamishoyosan (KSS) reduces lipid accumulation. (**A**) Induction time course of 3T3-L1 cell differentiation. 3T3-L1 cells were seeded on a 24-well plate at 2 × 10^4^ cells per well and cultured for 3 days. After confluent (0 day), 1 μM dexamethasone (Dex; D), 0.5 mM 3-isobutyl-1-methylxanthine (IBMX; M), and 10 μg/mL insulin (I) were added to induce differentiation for 3 days followed by additional 2 days culture with 5 μg/mL insulin (day 3–5). Thereafter, the medium was changed to normal Dulbecco’s modified Eagle’s medium (DMEM) every 2 days (day 5–8). Kampo medicines were administered at a concentration of 1 mg/mL after the onset of differentiation (day 0–8). (**B**) Images of Oil-Red-O staining of 3T3-L1 cells on day 8 are shown. (**Ba**) None (no DMI differentiation induction). (**Bb–f**) control and Kampo medicine administration groups ((**Bb**); H_2_O (**Bc**); KSS, (**Bd**); Hochuekkito (HET), (**Be**); Shoseiryuto (SST), (**Bf**); Goreisan (GRS) with DMI differentiation induction). The scale bar indicates 200 μm. (**C**) KSS suppresses lipid accumulation. (**Ca**) Oil-Red-O dye was extracted with isopropanol to measure intracellular lipid accumulation, and the absorbance was measured at 490 nm as compared with H_2_O (with DMI differentiation induction). (**Cb**) Cell viability after 8 days of culture was evaluated with the CCK-8 assay. Absorbance measured at 450 nm is shown as relative absorbance to H_2_O (with DMI differentiation induction). Data are shown as the mean ± standard deviation (*n* = 3). * *p* < 0.05 vs. H_2_O (with DMI differentiation induction).

The mouse 3T3-L1 preadipocyte cell line was obtained from the Japanese Collection of Research Bioresources Cell Bank (JCRB Cell Bank, Osaka, Japan). 3T3-L1 cells were cultured in high-glucose Dulbecco’s modified Eagle’s medium (DMEM; Sigma–Aldrich, St. Louis, MO, USA) containing heat-inactivated 10% fetal bovine serum (FBS; Thermo Fisher Scientific, Waltham, MA, USA), 30 U/mL penicillin (Meiji Seika Pharma, Tokyo, Japan), and 30 μg/mL streptomycin (Meiji Seika Pharma) at 37 °C in a humidified atmosphere of 5% CO_2_. For adipocyte differentiation, cells were seeded on a 24-well plate at a density of 2 × 10^4^ cells per well using the time course shown in Figure 1A. After reaching the confluence (0 days), adipocyte differentiation was initiated using the same medium, but supplemented with 1 μM Dex (Wako, Osaka, Japan), 0.5 mM IBMX (Wako), and 10 μg/mL insulin (Sigma–Aldrich) (hereafter DMI) for 3 days (day 0–3). The medium was then replaced with medium containing 5 mg/mL insulin for 2 more days (day 3–5) and then changed to fresh medium every 2 days (day 5–8). This differentiation induction method was termed the DMI method. 3T3-L1 cells were used up to the 4th passage to avoid cell phenotypic changes.

### 2.2. Preparation of Kampo Medicine and Composition of the Ten Herbs and Eight Major Components of KSS

Kamishoyosan (KSS), Hochuekkito (HET), Shoseiryuto (SST), and Goreisan (GRS) were kindly provided by Tsumura & Co. (Tokyo, Japan). Bupleuri Radix (BR), Paeoniae Radix (PR), Atractylodes Lancea Rhizoma (ALR), Angelicae Radix (AR), Poria (PO), Gardeniae Fructus (GF), Moutan Cortex (MC), Glycyrrhizae Radix (GLR), Ingiberis Rhizoma (IR), and Menthae Herb (MH) were purchased from Tsumura & Co. The extraction method of each Kampo medicine was as follows. One hundred milligrams of the powder was dissolved in 1 mL medium. The solution was shaken at 200 rpm and 38 °C for 30 min in a model BR-22FH constant temperature shaker (Taitec, Saitama, Japan). The solution was then centrifuged at 14,000× *g* for 25 min using a model 5922 device (Kubota, Tokyo, Japan). The supernatant was sterilized by using a 0.22-μm pore size membrane filter (Thermo Fisher Scientific). The eight major components of KSS were dissolved in dimethyl sulfoxide (DMSO, Wako). These compounds were: saikosaponin B1 (SSb1, Wako), saikosaponin B2 (SSb2, LKT Laboratories, St. Paul, MN, USA), paeoniflorin (PNF, LKT), β-eudesmol standard (EU, Wako), geniposide (GEN, Wako), paeonol (PN, LKT), glycyrrhizic acid (GA, Combi-Blocks, San Diego, CA, USA), and 6-shogaol (SG, Cayman Chemicals, Ann Arbor, MI, USA). L-glutamine added to the phenol red-free DMEM was purchased from Nissui pharmaceutical (Tokyo, Japan), and sodium pyruvate solution was purchased from Wako. Glucocorticoid receptor inhibitor of RU-486 (mifepristone) was purchased from Tokyo Chemical Industry (Tokyo, Japan).

### 2.3. Evaluation of Intracellular Lipid Accumulation

Cells were stained with Oil-Red-O (Sigma–Aldrich) as described previously [30]. 3T3-L1 cells were plated on a 24-well plate and induced to differentiate using the DMI method described earlier. The cells were rinsed with phosphate-buffered saline (PBS), fixed with 4% paraformaldehyde (PFA; Wako) for 14 min, and then stained with 3 mg/mL Oil-Red-O (in 60% isopropanol) for 10 min at room temperature. After staining, cells were washed once with 60% aqueous isopropanol and twice with PBS. After washing, the cells were observed using an ECLIPSE Ti-U inverted microscope (Nikon, Tokyo, Japan). Cell images were captured with a CCD camera (digital sight DS-L3, Nikon). Additionally, after the dye was extracted for 10 min with isopropanol, the absorbance was measured at 490 nm using a DTX 880 Multimode Detector (Beckman Coulter, Brea, CA, USA). All experiments were performed at least three times.

### 2.4. Assessment of Cell Viability

To investigate the effect of Kampo medicine, herbs, and major components on the cell viability, a Cell Counting Kit-8 (CCK-8; Dojindo Molecular Technologies, Kumamoto, Japan) was used according to the manufacturer’s instructions. Absorbance was measured at 450 nm using the DTX 880 Multimode Detector. All experiments were performed at least three times.

### 2.5. Quantitative PCR (qPCR)

3T3-L1 cells were seeded in a four-well plate (Thermo Fisher Scientific). Total RNA was prepared using ISOGEN II (Nippon Gene, Tokyo, Japan). The total RNA concentration was determined by measuring the absorbance at 260 nm using a NanoDrop 2000c spectrophotometer (Thermo Fisher Scientific) and NanoDrop 2000/2000c Operating software, version 1.4.2. Using 1 μg of total RNA as a template, cDNA was synthesized by reverse transcription reaction using the SuperScript^®^ Vilo™ cDNA synthesis kit (Thermo Fisher Scientific). Reverse transcription was conducted with a G-STORM GS482 thermal cycler (Life Science Research, Somerton, UK). The expression level of various genes regulating adipocyte differentiation was measured in the CFX 96^TM^ Real-Time System (BIO-RAD, Hercules, CA, USA) with the KOD SYBR^®^ qPCR Mix (TOYOBO, Osaka, Japan). qPCR was performed at 98 °C for 2 m for the initial denaturing, followed by 45 cycles of 98 °C for 10 s, 61 °C or 67 °C for 10 s, and 68 °C for 30 s, using specific primers (Table 1). The results were determined using the ΔΔC_T_ method and are shown as the fold-change relative to the control after normalizing to the expression of the 14-3-3 protein zeta/delta (Ywhaz) gene. The primer sets of Ywhaz housekeeping gene was purchased from TaKaRa Bio (Shiga, Japan). All experiments were performed at least three times.

### 2.6. Luciferase Reporter Assay

Dual-luciferase reporter assays were conducted as described previously [34]. One day before transfection, 3T3-L1 cells were cultured in antibiotic-free and phenol red-free medium (Sigma–Aldrich) containing L-glutamine, sodium pyruvate, and 10% charcoal-dextran-stripped FBS (GE Healthcare, Salem, CT, USA). For electroporation, a mixture of 300 ng pGL 4.36 vector [luc2P, MMTV, Hygro] (Promega, Madison, WI, USA) and 3 ng internal standard pNL 1.1 PGK vector [Nluc PGK] (Promega) were used for 1.0–1.6 × 10^5^ cells with a MicroPorator MP-100 device (NanoEnTek, Seoul, Korea). The electroporation condition was two times voltage change to 1300 V for 20 ms. After transfection, cells were seeded on a 24-well plate and cultured in the antibiotic-free and phenol red-free DMEM containing 10% charcoal-dextran-stripped FBS. One day after electroporation, the medium was changed to the medium with Kampo medicines, herbs, or their components together with 1 μM Dex for 6 h. At 30 h after transfection, cells were collected using 100 μL of Reporter Lysis Buffer (Promega). The collected cell lysates were stored at −80 °C. GR-dependent luciferase activity was analyzed with the Dual-Luciferase^®^ Reporter Assay System kit (Promega). Emission intensity was measured using the GloMax^®^ Discover Microplate Reader GM3510 (Promega). The results were normalized to the internal pNL 1.1 PGK control and expressed as the fold-change of mean relative intensity. All experiments were performed at least three times.

### 2.7. Statistical Analysis

Results are expressed as mean ± standard deviation. Comparisons between groups were made using Student’s *t*-test or one-way analysis of variance (one-way ANOVA). *p* < 0.05 was considered statistically significant. Statistical analysis was performed using JMP^®^ Pro 14.2.0 (SAS Institute, Cary, NC, USA).

## 3. Results

### 3.1. KSS Reduces Intracellular Lipid Accumulation

Mouse 3T3-L1 cells, which are preadipocytes, were differentiated into adipocytes by DMI treatment. We examined the effects of KSS and the three Kampo medicines. Kampo medicines are composed of two to ten herbs. The number and volume of herbs are changed in the prescribed formulation depending on the intensity of the chief and indefinite complaints. The 129 kinds of Kampo medicines can be divided into two groups: those with and without Bupleuri Radix (BR). KSS contains BR and is prescribed for climacteric disorder and orofacial pain [35,36]. In this study, we examined the effects of Hochuekkito (HET), Shoseiryuto (SST), and Goreisan (GRS), as the control of KSS. HET contains BR, but its prescription spectrum is different from KSS. SST does not contain BR, and its spectrum is different from KSS. GRS does not contain BR but has a similar spectrum to KSS [18,37]. As shown in Figure 1A, KSS, HET, SST, or GRS (1 mg/mL) was added to DMEM together with DMI, and the cells were cultured for 8 days. Cells were stained with Oil-Red-O to assess lipid accumulation and the degree of differentiation [12]. Only KSS reduced the Oil-Red-O staining (Figure 1B). To compare the quantity of the accumulated lipid, we dissolved the accumulated Oil-Red-O in isopropanol and measured the absorbance. As shown in Figure 1Ca, KSS significantly reduced lipid accumulation. In addition, the CCK-8 assay showed that KSS, HET, SST, and GRS had no effect on cell viability at a concentration of 1 mg/mL (Figure 1Cb).

**Figure 2:**
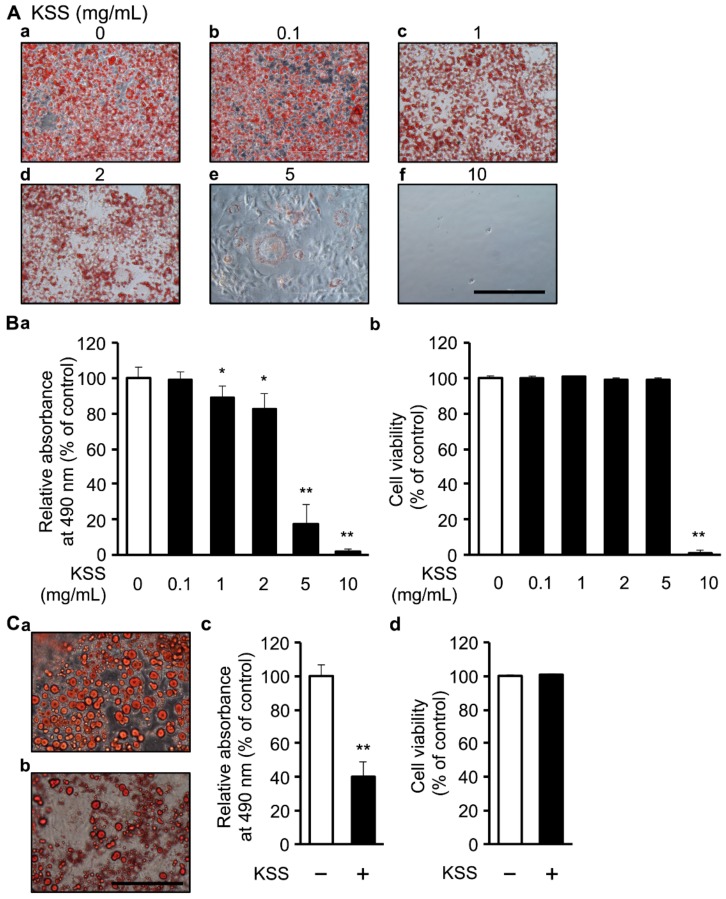
KSS reduces lipid accumulation in a dose-dependent manner. (**A**) KSS inhibited lipid accumulation in 3T3-L1 cells induced by the DMI method in a dose-dependent manner. Oil-Red-O staining images of cells cultured for 8 days is shown. (**Aa**) KSS 0 mg/mL, (**Ab**) 0.1 mg/mL, (**Ac**) 1 mg/mL, (**Ad**) 2 mg/mL, (**Ae**) 5 mg/mL, and (**Af**) 10 mg/mL. The scale bar indicates 200 μm. (**B**) KSS inhibited the lipid accumulation in 3T3-L1 cells cultured for 8 days in a dose-dependent manner. As in Figure 1, (**Ba**) the amount in lipid accumulation and (**Bb**) the cell viability was measured. (**C**) KSS suppressed lipid accumulation after 21 days of administration. (**Ca**,**b**) Oil-Red-O staining images of cultured cells are shown. (**Ca**) KSS (0 mg/mL) and (**Cb**) KSS (2 mg/mL). The scale bar indicates 200 μm. As in (**B**), (**Cc**) the amount of lipid accumulation was measured and is expressed as relative absorbance. (**Cd**) Cell viability. Data are shown as the mean ± standard deviation (*n* = 3). * *p* < 0.05 and ** *p* < 0.01 vs. H_2_O.

Next, we examined the dose-dependent effect of KSS. 3T3-L1 cells were differentiated for 8 days in the presence of 0.1, 1, 2, 5, or 10 mg/mL KSS. The cells were stained with Oil-Red-O. KSS reduced lipid accumulation (Figure 2A,Ba) without an appreciable change in cell viability at a concentration up to 5 mg/mL (Figure 2Bb). In the subsequent experiments, a concentration of KSS of 5 mg/mL or less was used.

Next, we examined the effects of KSS on lipid accumulation for longer periods. 3T3-L1 cells were differentiated for 21 days in the presence of 2 mg/mL KSS. KSS reduced the Oil-Red-O staining without affecting the cell viability (Figure 2Ca,b). KSS reduced the rate of lipid accumulation at 21 days more strongly than at 8 days (Figure 2Cc,d).

### 3.2. MC, Paeoniflorin, and Paeonol Inhibit Differentiation of 3T3-L1 Cells into Mature Adipocytes

**Figure 3:**
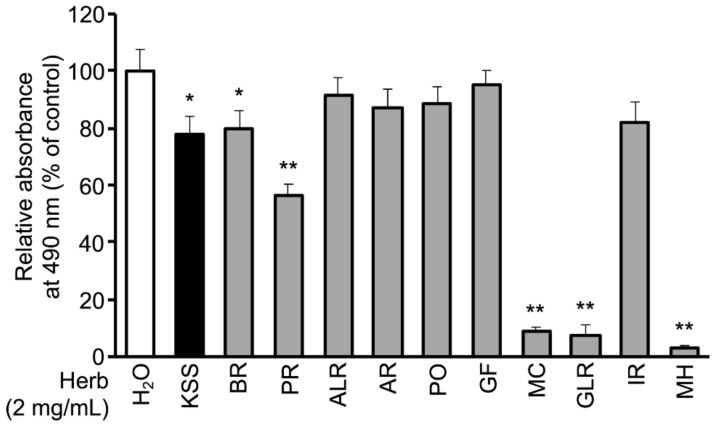
Bupleuri Radix (BR), Paeoniae Radix (PR), Moutan Cortex (MC), Glycyrrhizae Radix (GLR), and Menthae Herb (MH) in KSS reduce lipid accumulation. During differentiation induction by the DMI method, KSS or the 10 constituent herbs (2 mg/mL) were administered and cultured for 8 days. As in Figure 1, the amount of lipid accumulation was measured and is shown as relative absorbance. Data are shown as the mean ± standard deviation (*n* = 3). * *p* < 0.05 and ** *p* < 0.01 vs. H_2_O.

We sought to identify which of the 10 herbs of KSS suppressed the accumulation of lipids (Table S1). The 10 herbs of KSS were individually applied to cells at a concentration of 2 mg/mL together with DMI, and the cells were cultured for 8 days (Figure 1A). The cells were then stained with Oil-Red-O. BR, PR, MC, GLR, and MH significantly inhibited lipid accumulation (Figure 3 and Table S1). Next, we examined the dependence on their concentration. 3T3-L1 cells were differentiated for 8 days in the presence of 0.03, 0.1, 0.3, 1, or 2 mg/mL of PR, MC, GLR, and MH. All four herbs inhibited lipid accumulation in a dose-dependent manner (Figure S3Aa,Ba,Ca,Da), and the cell viability did not change greatly, except for in the group administered 2 mg/mL MH (Figure S3Ab,Bb,Cb,Db). Each herb contains several components. Furthermore, we aimed to identify the major components of KSS that suppress lipid accumulation. The eight major components of KSS were selected based on both the 3D-HPLC (Figure S1 and Table S2) and the KSS product management information reported by Tsumura Co. Among the eight major components, herb-derived components that inhibited differentiation were BR-derived saikosaponin B1 (SSb1) and saikosaponin B2 (SSb2); MC-derived paeoniflorin (PNF) and paeonol (PN); and GLR-derived glycyrrhizic acid (GA). Herb-derived components that had no effect on differentiation were ALR-derived β-eudesmol standard (EU), GF-derived geniposide (GEN), and IR-derived 6-shogaol (SG). We excluded MH-derived L-menthol from the examination because there was no information on the 3D-HPLC (Figure S1). One of the eight major components of KSS was applied to the cells together with DMI, and the cells were cultured for 8 days.

**Figure 4:**
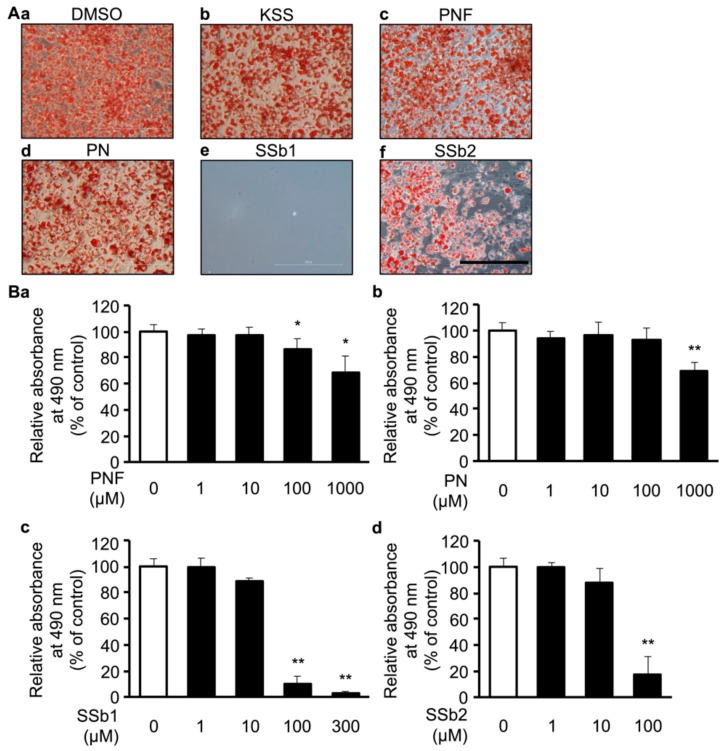
Paeoniflorin (PNF), Paeonol (PN), Saikosaponin B1 (SSb1), and Saikosaponin B2 (SSb2) reduce lipid accumulation in a dose-dependent manner. (**A**) (**Aa**) DMSO (DMI method only), (**Ab**) KSS 2 mg/mL, (**Ac**) PNF 1000 μM, (**Ad**) PN 1000 μM, (**Ae**) SSb1 300 μM, or (**Af**) SSb2 100 μM was added in culture media and cultured for 8 days. Images of Oil-Red-O staining are shown. The scale bar indicates 200 μm. (**B**) PNF, PN, SSb1, and SSb2 were administered during differentiation induction by the DMI method and cells were cultured for 8 days. As in Figure 1, the amount of lipid accumulation was measured and is shown as relative absorbance. (**Ba**) PNF, (**Bb**) PN, (**Bc**) SSb1, and (**Bd**) SSb2. Data are shown as mean ± standard deviation (*n* = 3). * *p* < 0.05 and ** *p* < 0.01 vs. DMSO.

The reduced number of Oil-red-O staining cell nodules observed in the treatment with 1000 μM PNF, 1000 μM PN, 300 μM SSb1, or 100 μM SSb2 compared with DMSO. Furthermore, Oil-Red-O staining was reduced in a dose-dependent manner (Figure 4A,B), but cells detached from the bottom by SSb1 and SSb2 within 2 days after the application (Figure 4A). SG at 30 μM reduced Oil-Red-O staining, 1000 μM GEN non-significantly reduced the red signal of Oil-Red-O staining, and 300 μM GA did not change the signal intensity (Figure S4A,B). EU at 1000 μM also reduced the red signal intensity, but cells detached from the bottom within 2 days after the application (Figure S4A). These results suggested that MC, PNF, and PN reduced lipid accumulation, and SSb1, SSb2, and EU were cytotoxic at the concentrations used. SSb1, SSb2, and EU were excluded from further studies.

### 3.3. KSS Inhibits Early Differentiation into Mature Adipocytes by Inhibiting the Action of Dex

**Figure 5:**
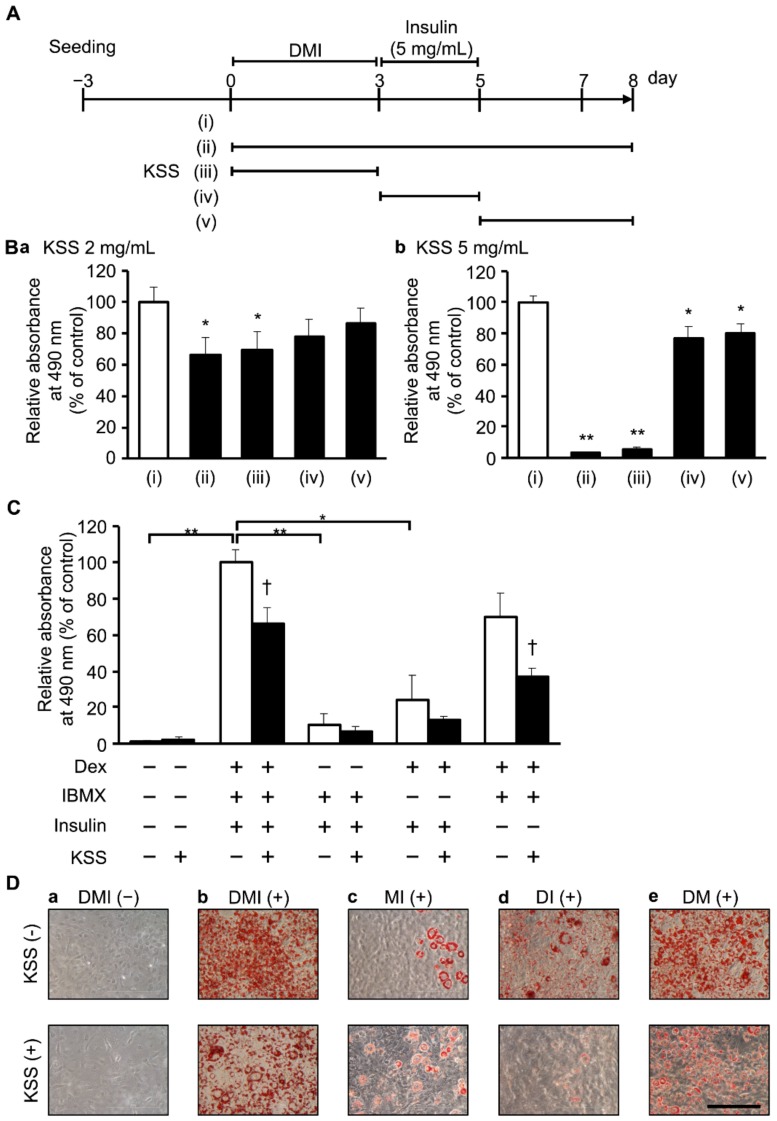
KSS inhibits early differentiation into adipocytes through the inhibition of Dex action. (**A**) 3T3-L1 cell differentiation stage and KSS administration time are shown. (i) No KSS administration, (ii) 0–8 days, (iii) 0–3 days (early-stage), (iv) 3–5 days (middle-stage), and (v) 5–8 days (late-stage). (**B**) KSS was administered at each differentiation stage. As in Figure 1, the amount of lipid accumulated at 8 days is measured and shown as relative absorbance. (**Ba**) KSS (2 mg/mL), (**Bb**) KSS (5 mg/mL). (**C**) 3T3-L1 cells were cultured for 3 days in the presence of two of the three types of differentiation inducers (1 μM Dex, 0.5 mM IBMX, and 10 μg/mL insulin). KSS (2 mg/mL) was administered for 8 days from the start of differentiation. The amount of lipid accumulated was measured as in Figure 1, and the relative absorbance is shown for the DMI method and KSS (-). (**D**) Oil-Red-O stained images of the cells cultured for 8 days. Upper: KSS (-), Lower: KSS (+), (**Da**) DMI (-), (**Db**) DMI (+), (**Dc**) MI (+), (**Dd**) DI (+), and (**De**) DM (+). The scale bar indicates 200 μm. Data are shown as mean ± standard deviation (*n* = 3). * *p* < 0.05 and ** *p* < 0.01 vs. (i) KSS (-) administration (**B**) or DMI method and KSS (-) (**C**). † *p* < 0.05 vs. KSS (-) (C).

In the process of differentiation of 3T3-L1 cells into mature adipocytes, various genes are expressed under strict regulation to form differentiation stages (early, middle, and late) (Jun do et al., 2011). To understand the differentiation stage at which KSS exhibited the inhibitory effect, 3T3-L1 cells were differentiated in the presence of 2 or 5 mg/mL KSS during 0–3 days (early stage), 3–5 days (middle stage), 5–8 days (late stage), and 0–8 days (Figure 5A). As shown in Figure 5B, the application of 2 mg/mL KSS during the early stage significantly reduced lipid accumulation (69.7 ± 11.3%, *n* = 3), similar to that during 0–8 days (66.7 ± 10.6%, *n* = 3). The inhibitory effect during the middle and the late stages was very low (77.5 ± 11.5%, *n* = 3 and 76.4 ± 8.0%, *n* = 3 for both). Next, we identified the target(s) of the inhibitory effect of KSS in the early stage of differentiation induced by DMI (Dex, IBMX, insulin). As shown in Figure 5C, Dex-deficient medium, IBMX-deficient medium, and insulin-deficient medium reduced lipid accumulation to 10.2 ± 6.2% (*n* = 3), 24.5 ± 13.1% (*n* = 3), and 70.3 ± 12.7% (*n* = 3), respectively, indicating that Dex and IBMX promote differentiation of 3T3-L1 cells. Moreover, 2 mg/mL KSS inhibited the lipid accumulation to 6.6 ± 2.8% (*n* = 3) in Dex-deficient medium, 13.5 ± 1.5% (*n* = 3) in IBMX-deficient medium, and 37.2 ± 4.3% (*n* = 3) in insulin-deficient medium. The reduction of the lipid accumulation by KSS was less in the Dex-deficient medium than in the IBMX-deficient medium, suggesting that KSS inhibits differentiation through Dex (Figure 5C,D).

### 3.4. KSS, MC, and Paeonol Alter the Expression of Adipocyte Differentiation Marker Genes

**Figure 6:**
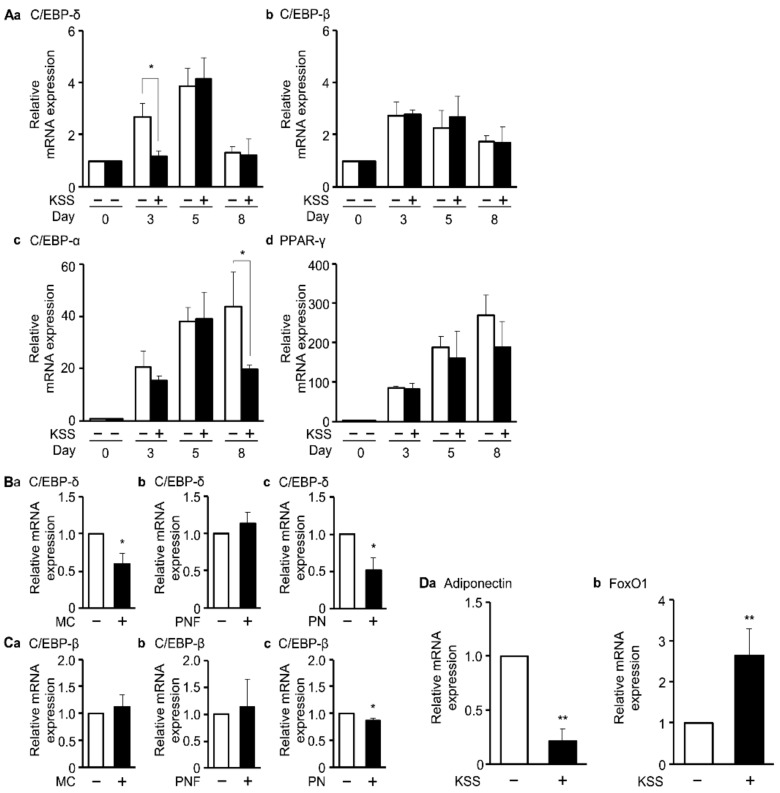
KSS, MC, and PN suppress C/EBP-δ gene expression. (**A**) KSS suppresses differentiation marker gene expression in a time-dependent manner. The gene expression level was quantified by the qPCR method. (**Aa**) C/EBP-δ, (**Ab**) C/EBP-β, (**Ac**) C/EBP-α, (**Ad**) PPAR-γ. (**B**,**C**) During differentiation induction, MC (1 mg/mL), PNF (1000 μM), and PN (1000 μM) were administered, and the expression level of the C/EBP-δ and C/EBP-β genes was quantified as in (**A**). (**a**) MC, (**b**) PNF, (**c**) PN. (**D**) Alteration of the expression level of (**Da**) Adiponectin and (**Db**) FoxO1 during differentiation induction with or without KSS. Data are shown as mean ± standard deviation (*n* = 3). * *p* < 0.05 and ** *p* < 0.01 vs. H_2_O or DMSO.

Since the expression levels of the genes of transcription factors C/EBP-δ, C/EBP-β, C/EBP-α, and PPAR-γ change during differentiation, they are considered to be differentiation marker in adipogenesis. We examined the expression of these four transcription factors by qPCR. KSS significantly suppressed gene expression of C/EBP-δ at differentiation day 3 (Figure 6Aa). Moreover, KSS suppressed gene expression of C/EBP-α, and PPAR-γ at differentiation day 8, no significant differences in the expression of C/EBP-β was observed (Figure 6Ab–d). Next, we examined the effect of MC and its constituents PNF and PN on the C/EBP-δ and C/EBP-β gene expression at differentiation day 3 (Figure 6B,C). MC at 1 mg/mL and 1000 µM PN suppressed gene expression of C/EBP-δ (Figure 6Ba,c). In addition, PN suppressed gene expression of C/EBP-β (Figure 6Cc). These results suggest that KSS, MC, and PN suppressed gene expression of C/EBP-δ at differentiation day 3. Supportively, the expression of adiponectin, a mature adipocyte marker and a secretion factor, was also diminished by KSS (Figure 6Da). On the other hand, KSS promoted the expression of FoxO1, that is an early marker of adipocyte differentiation (Figure 6Db).

### 3.5. KSS, MC, Paeoniflorin, and Paeonol Inhibit Promoter Activity of GR

**Figure 7:**
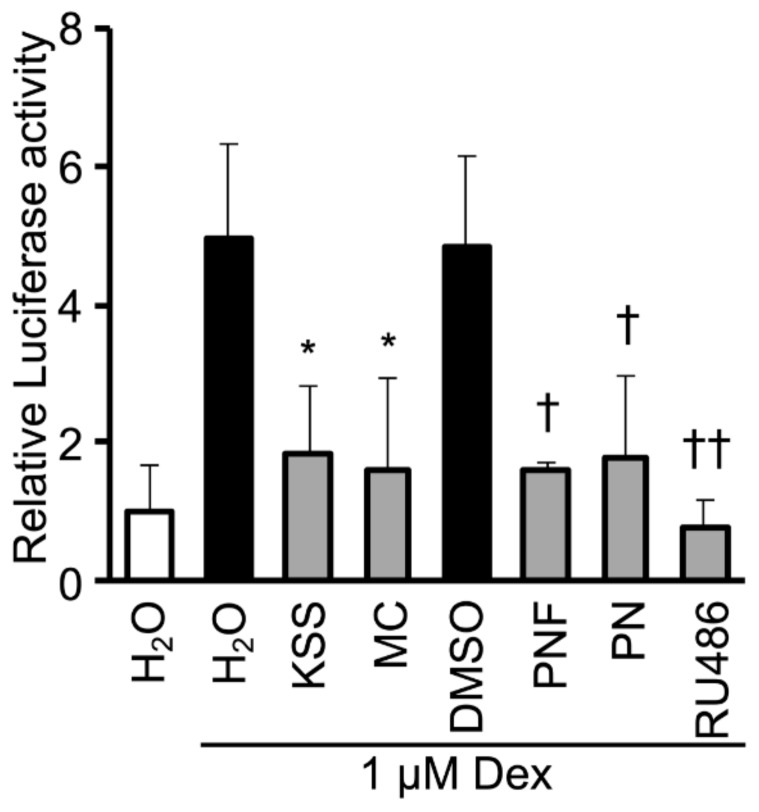
KSS, MC, PNF, and PN inhibit GR promoter activity. In the presence of 1 μM Dex, 2 mg/mL KSS, 1 mg/mL MC, 1000 μM PNF, 1000 μM PN, or 10 μM RU486 was administered, and a luciferase reporter assay was performed. Dex was used as a positive control for the GR promoter activity, and the relative activity was analyzed. Data are shown as the mean ± standard deviation (*n* = 3). * *p* < 0.05 vs. H_2_O (Dex added). † *p* < 0.05 and †† *p* < 0.01 vs. DMSO (Dex added).

Dex is involved in C/EBP-δ gene expression through the activation of the glucocorticoid receptor (GR) [38]. We investigated the effects of KSS, MC, PNF, and PN on the GR activity by luciferase reporter assay. The GR inhibitor RU486 was used as a GR antagonist [39]. KSS and MC inhibited Dex-induce GR promoter activity (Figure 7). Similarly, PNF and PN inhibited GR promoter activity, but the activity was weaker than that observed with RU486.

## 4. Discussion

**Figure 8:**
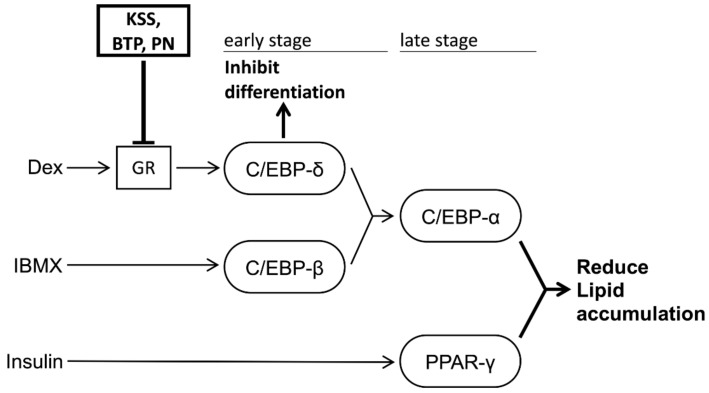
KSS, MC, and PN suppress lipid accumulation by suppressing Dex-induced GR activation and controlling early differentiation in 3T3-L1 cells. 3T3-L1 cells are differentiated into mature adipocytes by the addition of Dex, IBMX, and insulin (DMI method). As a result of differentiation, inducers bind to the receptor and the signals are transferred to the nucleus. KSS, MC, and PN inhibit the promoter activity of GR induced by Dex. This suppression of the promoter activity reduces the expression of the transcription factor C/EBP-δ gene and consequently inhibits the initial differentiation of 3T3-L1 cells. By suppressing early differentiation, the expression levels of C/EBP-α and PPAR-γ genes are reduced, and lipid accumulation is suppressed.

In this study, we demonstrated that KSS suppressed C/EBP-δ expression by inhibiting Dex-induced GR promoter activity at the early stage of differentiation and, consequently, delayed differentiation into mature adipocytes as summarized in Figure 8. Kampo medicines are complex drugs composed of many herbs. The herbs have many components. The effects of Kampo medicines have not previously been analyzed hierarchically. We systematically analyzed the reduction of lipid accumulation caused by Kampo medicines, their herbs, and their major components. KSS inhibited the preadipocyte differentiation into mature adipocytes, but HET, SST, and GRS did not (Figure 1 and Figure 2). MC was identified as an effective herb among the 10 constituent herbs of KSS (Figure 3 and Figures S2 and S3). Finally, PN was shown to be an effective major component among eight MC-derived components (Figure 4 and Figure S4). In addition to KSS, MC, and PN inhibited Dex-induced GR activity (Figure 6 and Figure 7).

PN, an MC-derived ingredient, reduces lipid accumulation in the livers of high-fat diet-induced diabetic mice and improves glucose and lipid metabolism by increasing the phosphorylation level of Akt and expression of glucokinase and low-density lipoprotein receptor in human liver cancer-derived HepG2 cells [29]. In this study, PN reduced lipid accumulation by suppressing early differentiation into mature adipocytes by inhibiting Dex-induced GR promoter activity (Figure 4, Figure 6 and Figure 7) and the expression of the PPAR-γ gene, which is one of the late-stage markers of adipocytes. We hypothesize that PN-mediated anti-adipogenetic activity diminishes the phosphorylation of Akt by blocking GR promoter activity. Clarifying this detailed mechanism would be interesting for a better understanding of the function of PN in developing mature adipocytes.

KSS as well as PN inhibited the activation of GR by Dex, which was followed by the suppression of C/EBP-δ gene expression. GR knockdown reduced lipid accumulation in adipocytes at day 7, even in the presence of insulin [40]. However, lipid accumulation and expression of the adipogenetic marker genes were intact in GR knockdown adipocytes at day 21 with the continuous supplementation of insulin [40]. KSS as well as PN reduced lipid accumulation at the late stage of differentiation (5–8 days) (Figure 5), similar to the results in the GR knockdown adipocyte model. However, KSS reduced lipid accumulation at day 21 more than that on day 8 (Figure 2). Since KSS was added at the late-stage of differentiation, it is possible that mechanisms other than suppression of Dex and GR might be involved in the long-term administration of PN.

One limitation of our study was that we could not quantify the content of the major components in the KSS extract supernatant that was used. Therefore, each drug was administered at the maximum dose at which the drug did not affect cell viability was administered. Kampo medicines and herbal medicines exert drug actions after ingestion. Information on the absorption-distribution-metabolism-excretion of Yokukansan is available [41], but there is no information on the absorption-distribution-metabolism-excretion of KSS. The blood concentration of PN, one of major components of KSS, after oral intake, is very small [42]. We will analyze the bioavailability of PN using an in vivo system and optimize the proper concentration of KSS needed to exert anti-adipogenetic activity in a future study.

## 5. Conclusions

In conclusion, our results demonstrate that KSS can inhibit the early stages of differentiation in mouse adipocytes and suppress lipid accumulation in 3T3-L1 cells. Paeonol is a critical ingredient that contributes to the anti-adipogenetic effect of KSS by blocking glucocorticoid receptor activity in lipid-metabolizing cells. The effects of Kampo medicine and herbal medicines have not been hitherto analyzed hierarchically. In this study, we identified the effects of the ingredients.

## Figures and Tables

**Table 1:** Primer sequences for qPCR.

Gene	Sense	Antisense	Ref.
C/EBP-α	5′-CAAGAACAGCAACGAGTACC-3′	5′-GTCACTGGTCAACTCCAGCAC-3′	[31]
C/EBP-β	5′-ACGACTTCCTCTCCGACCTC-3′	5′-CGAGGCTCACGTAACCGTAG-3′	
C/EBP-δ	5′-CTGCCATGTACGACGACGAGAG-3′	5′-GCTTTGTGGTTGCTGTTGAAGA-3′	
PPAR-γ	5′-CTGATGCACTGCCTATGAGC-3′	5′-TCACGGAGAGGTCCACAGAG-3′	
Adiponectin	5′-GCACTGGCAAGTTCTACTGCAA-3′	5′-GTAGGTGAAGAGAACGGCCTTGT-3′	[32]
FoxO1	5′-ACGAGTGGATGGTGAAGAGC-3′	5′-TGCTGTGAAGGGACAGATTG-3′	[33]

## Supplementary Materials

The followings are available online at https://www.mdpi.com/2072-6643/12/2/309/s1. Figure S1: 3D-HPLC diagram of KSS, Figure S2: PR, MC, GLR, MH change in lipid droplets, Figure S3: PR, MC, GR, MH reduce lipid accumulation in a dose-dependent manner, Figure S4: EU, SG major components of KSS reduce fat accumulation in a dose-dependent manner, Table S1: Composition of ten herbs of KSS, Table S2: Eight major compounds of KSS.
