# Supplementary material for: Paeonol, an Ingredient of Kamishoyosan, Reduces Intracellular Lipid Accumulation by Inhibiting Glucocorticoid Receptor Activity in 3T3-L1 Cells
Source: Nutrients. 2020 Jan 24;12(2):309. doi: 10.3390/nu12020309 (PMC7071193; doi:10.3390/nu12020309)
Supplement: Supplementary file 1 [file nutrients-12-00309-s001.pdf]

Supplementary Materials:

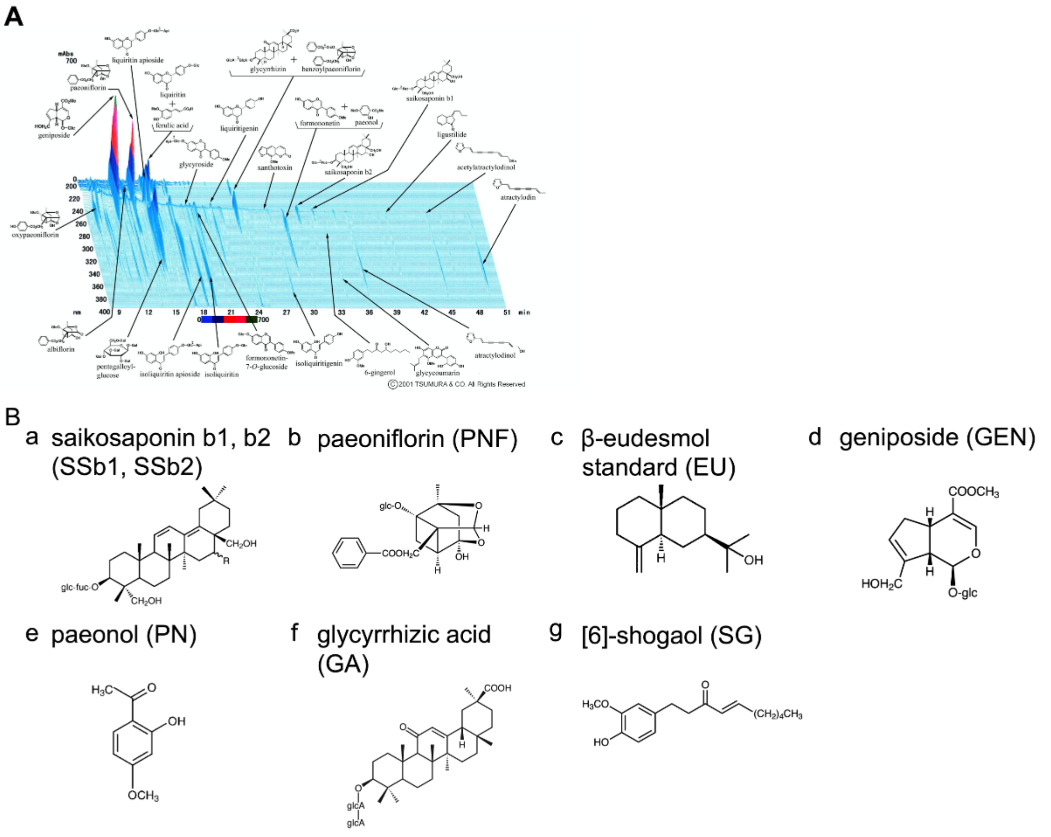

Figure S1. 3D-HPLC diagram of KSS. (**A**) 3D-HPLC results were kindly provided by Tsumura & Co. to identify components of KSS. The HPLC apparatus consisted of a Shimadzu LC-10A (analysis software: CLASS-M10A ver. 1.64, Tokyo, Japan) equipped with a multiple wavelength detector (wavelength range: 200–400 nm; Shimadzu SPD-M10AVP diode array detector) and an autoinjector (Shimadzu CTO-10 AC). The HPLC conditions were as follows: column, TSK-GEL 80TS octadecylsilyl column (internal diameter: 250 × 4.6 mm; TOSOH, Tokyo, Japan); eluent, (**i**) 0.05 M AcONH4 (pH 3.6), (**ii**) 100% CH3CN. A linear gradient of 90% (i) and 10% (ii) changing over 60 min to 0% (i) and 100% (ii) was used. Once a (ii) concentration of 100% had been achieved, it was maintained for 20 min (temperature, 40 °C; flow rate, 1.0 mL/min). (**B**) Eight major components of KSS (**Ba**) saikosaponin B1, B2 (SSb1, SSb2), (**Bb**) paeoniflorin (PNF), (**Bc**) β-eudesmol standard (EU), (**Bd**) geniposide (GEN), (**Be**) paeonol (PN), (**Bf**) glycyrrhizic acid (GA), (**Bg**) [6]-shogaol (SG).

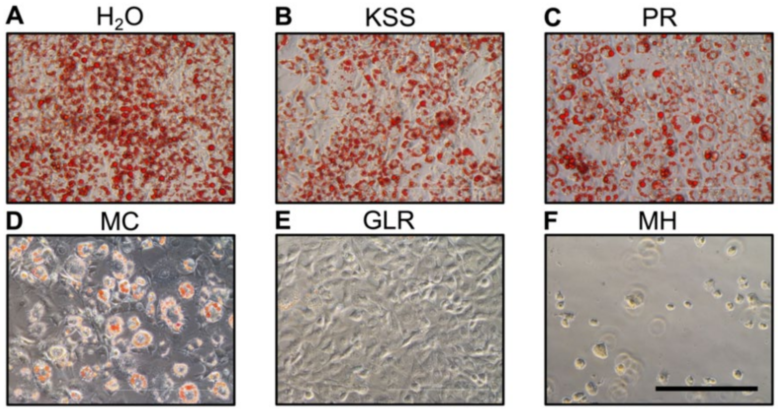

Figure S2. PR, MC, GR, MH change lipid droplets. (**A–F**) Oil-Red-O stained images of cells cultured with PR, MC, GR and MH for 8 days are shown. (**A**) H_2O (Control), (**B**) KSS 2 mg/mL, (**C**) PR 2 mg/mL, (**D**) MC 2 mg/mL, (**E**) GLR 2 mg/mL, (**F**) MH 2 mg/mL. The scale bar indicates 200 μm.

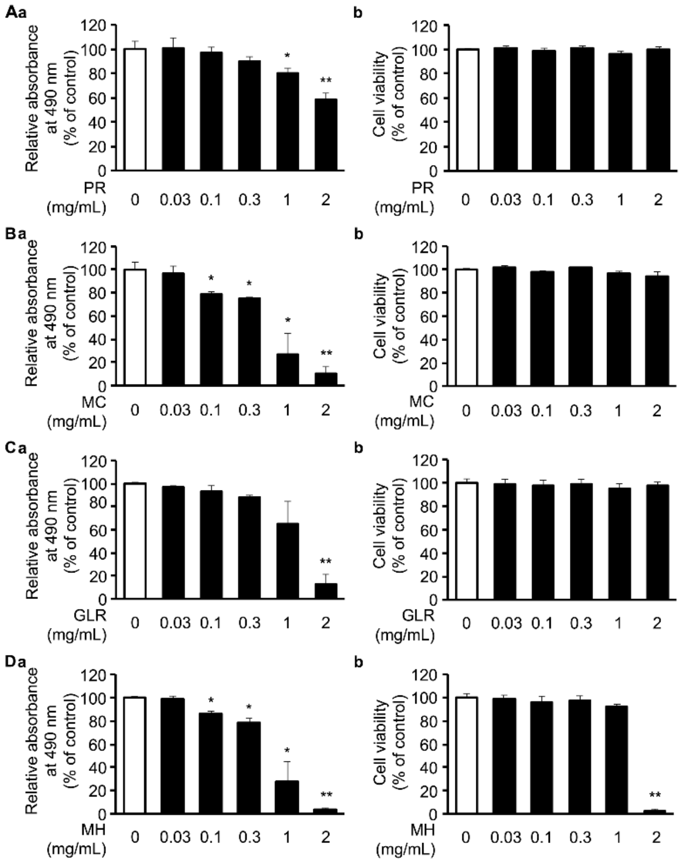

Figure S3. PR, MC, GR, MH reduce lipid accumulation in a dose-dependent manner. (**Aa**, **Ba**, **Ca**, **Da**) 3T3-L1 cells induced to differentiate using DMI method were administered (**A**) PR, (**B**) MC, (**C**) GLR, (**D**) MH, and cultured for 8 days did. As in Figure 1, the amount of lipid accumulation in the cells is measured and shown as relative absorbance. (**Ab**, **Bb**, **Cb**, **Db**) The cell viability is shown as in Figure 1. Data are shown as mean ± standard deviation (n = 3). * $p < 0.05$ and ** $p < 0.01$ vs. 0 mg/mL.

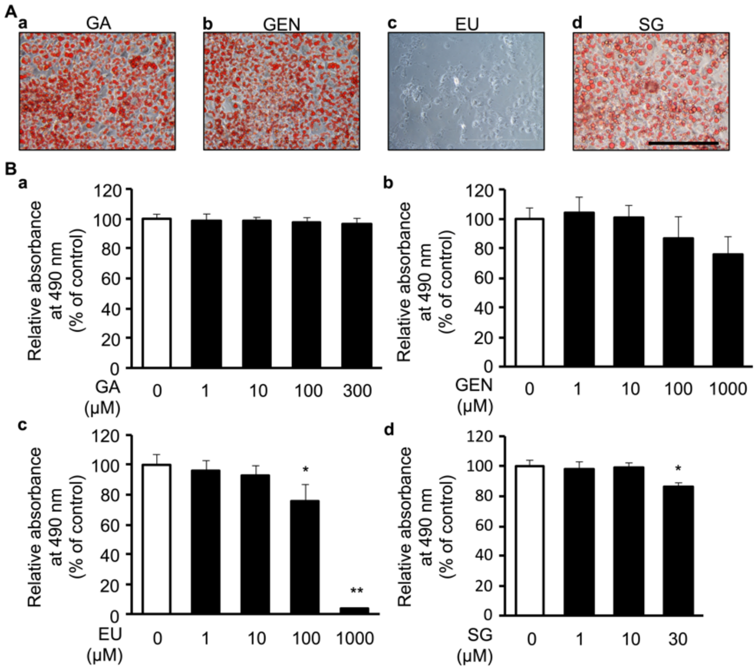

Figure S4. EU, SG major components of KSS reduce fat accumulation in a dose-dependent manner. (**A**) (**Aa**) GA 300 μM, (**Ab**) GEN 1000 μM, (**Ac**) EU 1000 μM and (**Ad**) SG 30 μM were administered during differentiation induction by the DMI method and cultured for 8 days. An Oil-red-O stained image is shown. The scale bar indicates 200 μm. (**B**) As in Figure 1, the amount of lipid accumulation in the cells is measured and shown as relative absorbance. (**Ba**) GA, (**Bb**) GEN, (**Bc**) EU, (**Bd**) SG. data are shown as mean ± standard deviation (n = 3). * $p < 0.05$ and ** $p < 0.01$ vs. 0 mg/mL.

Table S1. Ten Herbals of KSS.

Name	(g)	Abbreviation
Bupleuri Radix	3.0	BR
Paeoniae Radix	3.0	PR
Atractylodes Lancea Rhizoma	3.0	ALR
Angelicae Radix	3.0	AR
Poria	3.0	PO
Gardeniae Fructus	2.0	GF
Moutan Cortex	2.0	MC
Glycyrrhizae Radix	1.5	GLR
Ingiberis Rhizoma	1.0	IR
Menthae Herba	1.0	MH

Table S2. Eight Major Compounds of KSS.

Name	Component	Abbreviation
Bupleuri Radix	Saikosaponin B1	SSb1
	Saikosaponin B2	SSb2
Paeoniae Radix	Paeoniflorin	PNF
Atractylodes Lancea Rhizoma	β-Eudesmol standard	EU
Gardeniae Fructus	Geniposid	GEN
Moutan Cortex	Paeonol	PN
	Paeoniflorin	PNF
Glycyrrhizae Radix	Glycyrrhizic acid	GA
Ingiberis Rhizoma	6-Shogaol	SG
